# The Influence of a Christian, Seventh-Day Adventist Lifestyle Based on Religious Principles on the Risk of Developing Type 2 Diabetes Mellitus

**DOI:** 10.3390/healthcare13162044

**Published:** 2025-08-19

**Authors:** Maricel Herrera, Cristabel Grados, Salomon Huancahuire-Vega

**Affiliations:** 1 Human Medicine School, Universidad Peruana Unión (UPeU), Lima 15464, Peru; giraleyherrera@upeu.edu.pe (M.H.); cristabelgrados@upeu.edu.pe (C.G.); 2 Public Health, Graduate School, Universidad Peruana Unión (UPeU), Lima 15464, Peru

**Keywords:** lifestyles, type 2 diabetes mellitus, nutritional habits, spirituality, seventh-day adventists

## Abstract

Background: Type 2 Diabetes Mellitus (T2DM) is a growing global health concern, closely linked to modifiable lifestyle factors. Emerging evidence suggests that religious principles can shape behaviors that promote physical and mental well-being. Objective: This study explores how adherence to a lifestyle rooted in Seventh-Day Adventist beliefs may influence the risk of developing T2DM. Methods: This study is of a non-experimental quantitative design with a cross-sectional and explanatory scope. The sample consisted of 303 participants (adults who had been active Seventh-Day Adventist Church, SDA, members for the past 3 years), selected by non-probabilistic convenience sampling. Data were collected using the Adventist Lifestyle questionnaire (eight dimensions) and the FINDRISC scale. The non-parametric statistics, Rho Spearman, and ordinal logistic regression analysis were used. Results: The results showed that lifestyle levels based on religious principles significantly predicted the risk of developing T2DM (X^2^ = 100.34, *p* < 0.05). The model explained approximately 31% of the variance in T2DM risk (R^2^ = 0.31), indicating a moderate association between lifestyle and risk. Specifically, individuals with lower (less healthy) lifestyle scores had a significantly higher likelihood of being classified in higher risk categories for T2DM. It was also observed that there is a significant relationship between the risk of developing T2DM and the following dimensions: Rest (rho = −0.16, *p* < 0.05), exercise (rho = −0.13, *p* < 0.05), temperance (rho = −0.19, *p* < 0.05), nutrition (rho = −0.66, *p* < 0.05) and spirituality (rho = −0.57, *p* < 0.05). Conclusions: A healthier lifestyle promoted by religious principles, such as the SDA, reduces the risk of developing T2DM. A stronger correlation was perceived between nutrition and spirituality since these practices are related to a low risk of developing the disease.

## 1. Introduction

T2DM is a metabolic disease that affects diverse people globally, regardless of economic strata or demographic groups. It is generated by the pancreas’s deficient production of the hormone insulin or its progressive misuse. This causes dysregulations in the metabolism of carbohydrates and consequently leads to constantly high glucose levels in the blood [1,2].

According to the World Health Organization (WHO), in 2019, diabetes was the direct cause of 1.5 million deaths worldwide [3]. In the same year, the Pan American Health Organization (PAHO) referred to a number close to 62 million people in the Americas (422 million people worldwide) who have T2DM, most of whom live in countries with medium to low socio-economic conditions. It also indicates that 244,084 deaths are directly assigned to this disease each year [4].

The increase in T2DM cases could be a consequence of lifestyle, which is characterized by an unhealthy diet, the absence of or little physical activity, smoking, alcohol consumption, overweight or obesity, high blood pressure, hypercholesterolemia, and risk factors that cannot be modified, such as age, sex, and genetic aspects [5]. Some of these factors increase the risk of insulin resistance, leading to T2DM [6].

Therefore, the population must know and adopt healthy lifestyles encompassing different dimensions of human well-being, considering the body and the mind. These approaches are comprehensive and interrelated to form a balanced life [7]. Religious principles can significantly influence the practice of a healthy lifestyle, as certain spiritual traditions promote habits that favor physical, mental, and social well-being [8].

Based on biblical principles, the Seventh-Day Adventist Church (SDA) promotes a comprehensive healthy lifestyle among parishioners, focusing on caring for the body as a “temple of the Holy Spirit” [9]. This approach, known as the Adventist health message, includes practices that positively impact physical, mental, emotional, and spiritual health [10]. The aspects covered by these practices include adequate water consumption, rest, physical exercise, exposure to sunlight and pure air, nutrition, temperance, and spirituality [11]. The practice of this lifestyle is associated with these religious principles, which could contribute positively to the physical and mental health of the population, being associated with a lower risk of developing non-communicable diseases such as heart disease and T2D, among others [12]. Due to these considerations, this study aims to determine if the lifestyle governed by the religious principles of the SDA influences the decrease or increase in the risk of developing T2DM.

## 2. Materials and Methods

### 2.1. Type of Study

The study is a quantitative, non-experimental research design, with a cross-sectional and explanatory scope. This design was chosen because it allows for the analysis of relationships between variables at a specific point in time without manipulating them, which is appropriate given the ethical and practical limitations of experimenting with human participants.

### 2.2. Study Population and Sample

The study population consisted of members baptized in the SDA who were over 18 years of age and who attended and actively participated. To collect the information, the pastoral area of the main SDA of the Lima East region was investigated using the parishioners’ statistics, confirming the continuous active presence of an average of 3000 adult attendees. The Simple Random Sampling Calculator program calculated the sample size in 341 adult members, with a 95% CI and 5% relative error. The sampling was non-probabilistic and convenient. The inclusion criteria were Adventist participants who agreed to participate in the study. Participants with a diagnosis of T2DM, minors, and those who did not wish to participate in the research were excluded. Finally, the sample comprised 303 participants, excluding 38 people (11%) by the above criteria. The raw data are provided as a Supplementary File.

### 2.3. Procedures and Instruments

The data collection technique was conducted in person through physical questionnaires and the Google Forms platform was used for those who preferred conducting it online. As instruments, we use 2 questionnaires: FINDRISC and Adventist Lifestyle. Data collection was carried out from September to December 2024.

The first instrument used for the independent variable, lifestyle, was the “Adventist Lifestyle” questionnaire, which includes the 8 dimensions promoted by the SDA. This instrument was validated by expert judgment in 2016, obtaining a construct validity: sig = 0.000 < α (0.05). Bartlett’s measurement is at a considerable level of 0.722, and Cronbach’s Alpha reliability is at 0.909. It contains 66 items: Water Intake, Rest, Exercise, Exposure to Sunlight, Air, Nutrition, Temperance, and Spirituality, with an ordinal scale rating in the Likert model in a positive direction and 21 questions in the reverse direction. Each dimension is assessed by multiple items scored on a 5-point Likert scale (0 = never, 4 = always). The lifestyle levels were made based on the percentile cuts of 25 and 75, reaching 5 categories, where the lifestyle score was divided into Critical: 148–174 points, deficient: 175–201, at risk: 202–227, acceptable: 228–254 and healthy: 255–281 [13].

For the dependent variable, the risk of developing T2DM, the FINDRISC (Finnish Diabetes Risk Score) instrument was used, which determines the risk of developing T2DM [14]; in 2014, it was adapted and validated for the population of Peru by the Ministry of Health. It was included in the “Clinical Practice Guide for the Prevention, Diagnosis, Treatment and Control of T2DM.” It contains 8 questions and their respective answers with categories that involve “age, BMI, waist circumference, physical activity, daily consumption of fruits and vegetables, personal history of treatment with antihypertensive, history of high blood sugar, and family history of T2DM”. For the results, they are divided into “low risk (<7), slightly elevated (7 to 11), moderate (12 to 14), high (15 to 20), and very high (>20)” [15].

### 2.4. Statistical Analysis

The surveys and data were processed using the software IBM SPSS Statistics version 29 (Statistical Package for Social Sciences), and then the cleaning of data and missing values was performed, and the items were grouped according to dimensions and variables. The descriptive statistical analyses were performed using frequency measures and cross tables. The normality of the quantitative variables was assessed using the Kolmogorov–Smirnov test, which indicated that the data did not follow a normal distribution (*p* < 0.05 for all variables). Given the ordinal nature of the lifestyle and risk variables, and the non-normal distribution of the data, non-parametric statistical methods were used. Specifically, Spearman’s Rho correlation coefficient was employed to assess the strength and direction of the association between lifestyle dimensions and the estimated risk of developing T2DM. This method does not assume linearity or normal distribution and is more appropriate for the data structure than Pearson’s correlation or linear regression. In addition, the ordinal logistic regression analysis was performed to determine the influence of overall lifestyle level on the probability of being classified into higher T2DM risk categories, as assessed by the validated FINDRISC tool. Because ordinal logistic regression does not yield a true coefficient of determination, we assessed model fit using Nagelkerke’s R^2^, an adjusted form of the Cox–Snell R^2^ that rescales values to range from 0 to 1 and serves as an approximate indicator of the proportion of variance explained by the model. This model is suitable for ordinal outcome variables and allows for the evaluation of the effect of a predictor on ordered risk categories.

### 2.5. Ethical Aspects

This research was submitted to the Ethics Committee of the Faculty of Health Sciences at Universidad Peruana Unión for evaluation and received ethical approval on 20 June 2023. This study was authorized under approval code 2022-CE-FCS-UPeU-055/2023 for its subsequent implementation. Likewise, the SDA Villa-Unión was requested to authorize the parishioners to access, complete, and submit the questionnaires. In addition, participants signed an informed consent form to use the required data, obtaining the corresponding information from the surveys and their importance. The application of the surveys was totally anonymous and voluntary. The methodology and informed consent were carried out following Helsinki’s Declaration.

## 3. Results

Table 1 shows the characteristics of the study sample. In total, 303 people participated, of which 50% (152) were adults aged 36 to 65 and 9% (26) were over 65. On the other hand, 48% (146) had experienced more than 20 years of Adventism. Finally, 11% of participants who met the exclusion criterion were excluded and not considered in the total sample.

Table 2 shows the levels of the study variables, where it is perceived that 27.8% of the sample have a healthy lifestyle and 15.8% present a critical lifestyle. In terms of the physical exercise dimension, 25.2% of the sample perform poorly compared to 18.8% who maintain a healthy lifestyle by exercising actively daily, and concerning the nutrition dimension, 33.4% support healthy and balanced nutrition. On the other hand, 23.8% of the sample present an acceptable state for the spirituality dimension of the SDA attendees. Regarding the levels of the risk of T2DM, Table 2 shows that 19.1% of the study sample lead a healthy lifestyle and, therefore, are at a low risk of developing T2DM. In comparison, 0.7% have very high levels of the risk of developing T2DM and an inadequate lifestyle.

Although the objectives corresponded to an influence analysis, it should be noted as a requirement that there was a significant relationship between the study variables, so the Kolmogorov–Smirnov normality test was first performed to know if the sample of 303 had a normal distribution of data. All the variables obtain a *p*-value < 0.05, which confirms that, in the study population, there is no normal distribution of data, so for the correlation analysis, a non-parametric statistic is used, Spearman’s rho, where it is observed (Table 3) that there is a moderate, inverse, and significant relationship (rho = −0.62 **, *p* < 0.05) between the levels of lifestyles and the levels of risk of developing T2DM in members of the SDA. As for the correlations with the dimensions of lifestyles, there is no significant relationship between the risk of developing T2DM and the dimensions water consumption (rho = −0.01, *p* > 0.05), sunlight (rho = −0.08, *p* > 0.05) and air (rho = −0.08, *p* > 0.05); that is, the development of this pathology is unrelated to the aforementioned healthy practices. The opposite occurs with the other dimensions since it is observed that there is a weak, inverse and significant relationship between the development of T2DM and rest (rho = −0.16 **, *p* < 0.05), exercise (rho = −0.13 *, *p* < 0.05) and temperance (rho = −0.19 **, *p* < 0.05). Likewise, a moderate, inverse and significant relationship is perceived between the risk of developing T2DM and nutrition (rho = −0.66 **, *p* < 0.05) and spirituality (rho = −0.57 **, *p* < 0.05); that is, as lifestyle levels improve in the dimensions above, the risk of developing T2DM decreases.

According to the results of the ordinal logistic regression model, which evaluated the effect of lifestyle levels within an Adventist Christian framework on the risk of developing T2DM, the model was statistically significant (X^2^ = 100.34, *p* < 0.05). This indicates that lifestyle levels are significantly associated with T2DM risk categories. The model explained approximately 31% of the variability in the risk of developing T2DM (Nagelkerke R^2^ = 0.31). An inverse association was observed, meaning that lower (less healthy) lifestyle scores were associated with higher predicted risk levels for T2DM. The results of the prediction values and ORs are presented in Table 4. Specifically, people with a critical lifestyle will be 51.8 times more likely to develop T2DM (β −3.95, *p* < 0.05). It is also observed that those with a poor lifestyle are exposed to the risk of developing T2DM 8.3 times more than usual (β −2.12, *p* < 0.05). Similarly, those who have a lifestyle at risk are 6.8 times more likely to generate this disease (β −2.12, *p* < 0.05); on the other hand, people who have an acceptable lifestyle are likely to decrease the risk of developing T2DM by 4.75 times (β −1.56, *p* < 0.05).

## 4. Discussion

Based on the results obtained, the model explains approximately 31% of the variance in T2DM risk, indicating a moderate association between lifestyle and risk; it is also observed that there is a probability that said risk increases by 51.8 times if people have critical lifestyle levels, and the opposite occurs with those who have an acceptable lifestyle because they are likely to decrease the risk of developing T2DM by 4.78 times. Therefore, while there are adequate levels in the practices of rest, exercise, nutrition, temperance, and spirituality, there are low levels of risk and vice versa. A study on 3475 Hispanic/Latino Adventists reported that plant-based eating results in a lower BMI than the diet of non-vegetarians [16]. Dr. Serena Tonstad et al. discuss (regarding vegetarian and non-vegetarian diets) the risk of developing T2DM according to the Adventist lifestyle in 22,434 men and 38,469 women from North America, where the BMI was lower in vegans, and a higher prevalence of T2DM in the non-vegetarian group was reported (7.6%) [17,18]. Consequently, the studies mentioned above indicate that the most effective way to prevent the development of T2DM is by rectifying the risk factors related to unhealthy lifestyles.

No significant relationship was found concerning the water consumption dimension; this variable and T2DM risk were disassociated [19]. Likewise, Johannes Naumann et al. reviewed 1139 articles and concluded that there was little evidence of the positive effects of water consumption on the development of T2DM [20]. However, in another study on French participants, water intake < 0.5 L/day was associated with a greater risk of developing T2DM, which conflicts with what was obtained in the present study; a possible explanation for this is the smaller sample size and type of population, in addition to the fact that the development of T2DM is more greatly linked to other factors [21].

Regarding the resting dimension, rho = −0.16 **, *p* < 0.05, is evidenced, which indicates an inverse and significant relationship, meaning that if there are adequate levels of rest, there will be a low risk of developing T2DM and vice versa. Rest is a period of inactivity or a decrease in it to recharge physically and mentally. The types of rest are sleep, meditation, weekly rest days, and/or annual vacations [22]. One of the fundamental principles of the SDA is sabbatical rest, which allows human beings to disconnect from daily activities that usually have stressful components and connect with their creator. A similar result was found in another study, where it was concluded that the lowest prevalence of T2DM was consistently observed in the group that had adequate rest [23].

Similarly, in the dimension of physical exercise, it was evidenced that it is inversely and significantly related since the frequency of physical exercise decreases the risk of developing T2DM. In addition, exercise is part of the SDA lifestyle, demonstrating a healthy lifestyle and reducing the risk of developing T2DM since it improves insulin sensitivity and glucose metabolism [24]. In another study, Heredia-Morales et al. studied Mexican participants, concluding that physical exercise is a fundamental factor in preventing T2DM [25]. Additionally, it was shown that adherence to moderate-intensity exercises of 150 min per week prevented non-communicable diseases such as T2DM in Adventist African Americans [26].

Regarding the dimension of exposure to sunlight, it was corroborated that there is no significant relationship between both variables; that is, contact and exposure to sunlight are not linked to the development of said pathology. On the other hand, in a study on non-Christian people, it was observed that an increase in exposure to acute sunlight was related to decreased levels of preprandial insulin; a possible reason for this controversy is that the sample size was much higher in a different population [27].

It was corroborated that there is no significant relationship regarding the pure air dimension; that is, both variables are not developed in a linked manner. However, Maayan Yitshak et al. studied a population in the US., where they mention that a critical risk of T2DM was found with long-term air pollution exposure; on the other hand, most recent research is limited, still evaluating whether air exposure is related to said pathology [28].

On the other hand, it is observed that the nutrition dimension is moderately, inversely, and significantly related. As long as adults have healthy practices in their diet or an adequate balance of it, they will have less risk of developing T2DM. In two systematic review studies in Adventists from North America and Non-Hispanics, the health consequences of vegetarian and non-vegetarian diets were compared, discerning that people who chose not to consume red meat had a degree of protection against cardiovascular and metabolic diseases [29,30]. On the other hand, the consumption of fruits and whole grains had an inverse relationship to the development of T2DM, unlike a diet based on red meat, alcohol, sugary drinks, and processed foods [31].

Similarly, it is perceived that there is an inverse and significant relationship between the temperance dimension and the risk of developing T2DM. If adults have high levels of self-regulation/self-control, they will have a low risk of contracting this diagnosis. One study supports those mentioned above, indicating that a 0.6% decrease in the HbA1c of patients with DM was contemplated for each self-monitoring practice performed [31]. Similarly, Kimberly Gouveia et al. studied Brazilian people with T2DM, where sufficient knowledge of self-control practices positively interfered with self-efficacy for glycemic correction [32].

In the spirituality dimension, it is evident that there is an acceptable lifestyle with 23.8%, thus indicating that those people who maintain a constant spiritual life and adequate care of their mental health have less risk of being diagnosed with T2DM. Religiosity, which is usually measured as attendance at religious events, is connected to better physical health. There are likely explanations, such as a healthier lifestyle, greater social support from church members, and more positive emotions [7]. Likewise, in a study of a population with T2DM, it is concluded that anguish due to the lack of hope causes participants to have a glycemic imbalance, making them more prone to depression [33]. These studies agree with the results of the present study since religiosity influences better mental health and spirituality. Consequently, it decreases the risk of developing T2DM.

Finally, several studies suggest that the health effects of religiously motivated lifestyles differ by religious tradition. In general Christian populations, religiosity has been linked to better glycemic control and self-management: a study reported that people who believe in God and participate in church activities were associated with a substantial and independent reduction in diabetes incidence [34]. Evidence from Muslim communities is more limited. Most lifestyle research has been conducted in Western or Judeo-Christian settings, and the authors caution that the results cannot be generalized to Muslims because Islamic traditions (e.g., Ramadan fasting) may modify dietary and physical activity patterns [35]. These differences underscore the need to interpret the present findings, which show that adherence to a Seventh-Day Adventist lifestyle is associated with a lower risk of type 2 diabetes, in the context of specific religious practices and to encourage further research in diverse religious communities.

### Limitations

This study presents several limitations. First, the sample was limited to members of a single Seventh-Day Adventist congregation in Lima-East, which may affect the generalizability of the findings to other populations. Second, although demographic variables such as age, sex, educational level, and marital status were collected, they were not included as covariates in the final ordinal logistic regression model. This was due to the limited sample size, which reduced the statistical power for multivariable adjustment without risking model overfitting. As a result, potential confounding effects could not be fully controlled, and the observed associations should be interpreted with caution. Future studies with larger and more diverse samples are recommended to incorporate these variables into adjusted models to better isolate the effects of lifestyle on T2DM risk. Additionally, the cross-sectional design limits the ability to infer causality. Finally, the non-normal distribution of data required the use of non-parametric tests, which may be less sensitive than parametric alternatives.

## 5. Conclusions

This study shows an inverse and significant relationship between a healthy lifestyle, based on Christian Adventist principles, and the risk of developing T2DM. That is, a balanced lifestyle reduces the likelihood of suffering from T2DM. The importance of following the eight natural remedies of the SDA, which include rest, exercise, nutrition, temperance, and spirituality, is highlighted. In particular, nutrition, with an emphasis on legumes, vegetables, and nuts, the low consumption of red or processed meats, and spirituality, based on trust in a superior being and their respect, show a more significant compensation. This study invites the population to adopt these habits to prevent the development of T2DM.

## Figures and Tables

**Table 1:** Sociodemographic characteristics of the study participants.

Variable		n (303)	%
Age	Young Adulthood (18–35)	125	41.2
	Middle Adulthood (36–65)	152	50.2
	Late Adulthood (>66)	26	8.6
Sex	Female	178	58.7
	Male	125	41.3
Marital status	Single	147	48.5
	Married	134	44.2
	Divorced	9	3
	Widowed	13	4.3
Educational level	Elementary	16	5.3
	Secondary	81	26.7
	University	203	67
	No Education	3	1
Years of Adventist	3 to 4	33	10.8
	5 to 9 Years	39	12.9
	10 to 19 Years	85	28.1
	20 to More	146	48.2

**Table 2:** Distribution of participants across categories of lifestyle dimensions and corresponding risk levels for developing type 2 Diabetes Mellitus (T2DM).

**Variable**	**Lifestyle Levels**										**Total**	
	**Criticism**		**Deficient**		**At Risk**		**Acceptable**		**Healthy**			
	**n**	**%**	**n**	**%**	**n**	**%**	**n**	**%**	**n**	**%**	**n**	**%**
Lifestyle	48	15.8	54	17.8	57	18.8	60	19.8	84	27.8	303	100
Water consumption	14	4.6	77	25.4	101	33.4	78	25.7	33	10.9	303	100
Rest	73	24.1	62	20.5	49	16.1	69	22.8	50	16.5	303	100
Exercises	71	23.4	76	25.2	51	16.8	48	15.8	57	18.8	303	100
Sunlight	65	21.5	178	58.7	49	16.2	11	3.6	0	0	303	100
Air	18	5.9	54	17.8	52	17.2	170	56.1	9	3	303	100
Nutrition	41	13.5	54	17.8	47	15.5	60	19.9	101	33.4	303	100
Temperance	4	1.3	68	22.4	183	60.5	48	15.8	0	0	303	100
Spirituality	64	21.1	62	20.5	59	19.5	72	23.8	46	15.1	303	100
**T2DM Risk**	**Lifestyle Levels**											
	**Criticism**		**Deficient**		**At Risk**		**Acceptable**		**Healthy**		**Total**	
	**n**	**%**	**n**	**%**	**n**	**%**	**n**	**%**	**n**	**%**	**n**	**%**
Low risk	2	0.7	8	2.6	11	3.6	26	8.6	58	19.1	105	34.6
Minor risk	10	3.3	39	12.9	37	12.2	27	8.9	22	7.3	135	44.6
Moderate risk	3	1.0	7	1.3	8	2.6	7	2.3	4	1.3	29	9.5
High risk	31	10.3	0	0	1	0.3	0	0	0	0	32	10.6
Extreme risk	2	0.7	0	0	0	0	0	0	0	0	2	0.7
Total	48	16	54	16.8	57	18.7	60	19.8	84	27.7	303	100

**Table 3:** Spearman’s Rho correlation coefficients between lifestyle dimensions and risk of developing type 2 Diabetes Mellitus (T2DM).

Lifestyle	T2DM Risk	
	Rho	*p*
Total lifestyle	−0.62 **	0.000
Water consumption	−0.001	0.91
Rest	−0.16 **	0.007
Exercises	−0.13 *	0.03
Sunlight	−0.08	0.14
Air	−0.00	0.94
Nutrition	−0.66 **	0.000
Temperance	−0.19 **	0.001
Spirituality	−0.57 **	0.000

** The correlation is significant at the 0.01 level (2 queues). * The correlation is significant at the 0.05 level (2 queues).

**Table 4:** Logistic regression model showing association between levels of Adventist lifestyle and risk categories for developing type 2 Diabetes Mellitus (T2DM).

Lifestyles	Risk of Developing T2DM							
	X^2^	*p*	R^2^	B	*p*	95% CI for OR		
						Odds Ratio	Inferior	Superior
Models’ adjustment	100.34	0	-	-	-	-	-	-
Nagelkerke	-	-	0.31	-	-	-	-	-
Critical lifestyle	-	-	-	−3.95	00.000 *	51.84	3.12	4.78
Poor lifestyle	-	-	-	−2.12	00.000 *	8.30	1.35	2.88
Lifestyle at risk	-	-	-	−1.92	00.000 *	6.83	1.16	2.68
Acceptable lifestyle	-	-	-	−1.56	00.000 *	4.75	0.78	2.33

* The correlation is significant at the 0.01 level.

## Data Availability

The data of the current study are available as Supplementary Material.

## Supplementary Materials

The following supporting information can be downloaded at https://www.mdpi.com/article/10.3390/healthcare13162044/s1: Dataset.
